# Understanding blood development and leukemia using sequencing-based technologies and human cell systems

**DOI:** 10.3389/fmolb.2023.1266697

**Published:** 2023-10-10

**Authors:** Branco M. H. Heuts, Joost H. A. Martens

**Affiliations:** Department of Molecular Biology, Faculty of Science, Radboud University, Nijmegen, Netherlands

**Keywords:** transcription factors, eRNA, gene regulatory networks, single-cell sequencing, iPSC, functional genomics, big data, hematopoiesis

## Abstract

Our current understanding of human hematopoiesis has undergone significant transformation throughout the years, challenging conventional views. The evolution of high-throughput technologies has enabled the accumulation of diverse data types, offering new avenues for investigating key regulatory processes in blood cell production and disease. In this review, we will explore the opportunities presented by these advancements for unraveling the molecular mechanisms underlying normal and abnormal hematopoiesis. Specifically, we will focus on the importance of enhancer-associated regulatory networks and highlight the crucial role of enhancer-derived transcription regulation. Additionally, we will discuss the unprecedented power of single-cell methods and the progression in using *in vitro* human blood differentiation system, in particular induced pluripotent stem cell models, in dissecting hematopoietic processes. Furthermore, we will explore the potential of ever more nuanced patient profiling to allow precision medicine approaches. Ultimately, we advocate for a multiparameter, regulatory network-based approach for providing a more holistic understanding of normal hematopoiesis and blood disorders.

## 1 Introduction

Normal hematopoietic development unfolds as an ordered, multi-stage process, tightly regulated by a complex interplay of intrinsic factors and microenvironmental cues (Fujiwara et al., 2010; Morrison and Scadden, 2014; Olson et al., 2020; Pucella et al., 2020). Our conventional understanding of these intricate mechanisms delineates cell fate decision into two primary branches: the myeloid and lymphoid lineages (Pucella et al., 2020). The myeloid lineage has crucial roles in oxygen transport, hemostasis, and innate immunity. For instance, erythrocytes specialize in oxygen transport (Dunn et al., 2016), while megakaryocytes produce platelets essential for blood clotting, contributing to hemostasis (Bluteau et al., 2009; Periayah et al., 2017). Furthermore, the innate immune response is orchestrated by monocytes, neutrophils, eosinophils, basophils, and dendritic cells, collectively ensuring effective defense against pathogens and foreign substances (Chaplin, 2010). The innate immune system also exhibits adaptive characteristics to effectively respond to subsequent immune triggers, i.e., trained immunity (Netea et al., 2020). On the other hand, the lymphoid lineage takes charge of the antigen-specific and long-term immunological memory (Bonilla and Oettgen, 2010). This involves the specialized functions of B cells, T cells, and natural killer (NK) cells, which work together to recognize and eliminate specific pathogens and provide long-term immune protection.

Through different stages of life, human hematopoiesis undergoes distinct phases of development. Adult hematopoiesis refers to the ongoing process that occurs in the bone marrow throughout an individual’s lifetime (Pucella et al., 2020). It primarily involves the generation of mature blood cells from hematopoietic stem cells (HSCs) present in the bone marrow niche. In contrast, embryonic hematopoiesis occurs during the early stages of prenatal development, taking place in distinct anatomical sites, such as the yolk sac, fetal liver, and later the bone marrow (Julien et al., 2016). During embryonic hematopoiesis, the first wave of blood cells arises from mesodermal precursors and gives rise to primitive cell types, including primitive megakaryocytes and macrophages, as well as nucleated erythrocytes (Lim et al., 2013; Atkins et al., 2021). Through subsequent waves of hematopoiesis, definitive HSCs are developed that generate a diverse repertoire of blood cells (Julien et al., 2016). Thus, while adult hematopoiesis supports the continuous production of blood cells in adulthood, embryonic hematopoiesis lays the foundation for the establishment of the hematopoietic system during early embryonic stages.

Normal functioning of blood cell types can be disrupted leading to disease, such as leukemia, myelodysplastic syndromes (MDS), thrombocythemia, among other blood disorders. Different genetic perturbations, including deletions, insertions, and translocations, participate in the pathobiology of these diseases. Some of these represent monogenetic diseases that are linked to specific gene mutations, but others suffer from multiple genetic aberrancies, for example, leukemias (Prange et al., 2014). The latter is often seen as a consequence of the accumulation of mutations over time. Indeed, as individuals age, a more gradual accumulation of mutations within the HSC compartment arises and clonal variability reduces, a phenomenon called clonal hematopoiesis (CHIP) (Marnell et al., 2021). As a consequence, these cells can gain a survival advantage, potentially setting the stage for future complications. Furthermore, the microenvironment can co-participate in the pathobiology (Wei et al., 2008), directing lineage fate and causing cellular heterogeneity. Importantly, the accumulation of driver mutations and transformation of a normal cell into a leukemic cell, i.e., cell of origin, influences the patient’s chance of survival and treatment strategy (Horton et al., 2012; Krivtsov et al., 2012; Chopra et al., 2019). As such, the continuous deconvolution of hematopoietic clones and identification of driver events provides important insights for diagnostics, prognostic, and designing of therapeutic tools in the clinic.

Traditionally, blood cells are classified based on their morphological characteristics and surface markers using techniques such as flow cytometry and immunohistochemistry (Weissman and Shizuru, 2008; Lin and Goodell, 2011; Weeda et al., 2022), as well as by relying on genetic information. Nowadays, single-cell technologies have emerged as additional powerful tools, to enable the analysis of individual cells and the identification of rare cell populations within complex heterogeneous mixtures (Nathan et al., 2019). This allows us to gain further insights into the heterogeneity, dynamics, and developmental trajectories of blood cells as we now can incorporate multimodal approaches, such as combining transcriptomics with proteomics (Triana et al., 2021; Konturek-Ciesla et al., 2023), to uncover new biomarkers and molecular signatures that can classify blood cells with higher resolution. These developments would not have been possible without the advent of next-generation sequencing (NGS).

Indeed, NGS methods have deepened our understanding of normal and abnormal hematopoiesis on the basis of key regulatory pathways, gene expression profiles, and epigenetic patterns (Prange et al., 2014; Huang et al., 2016; Choi et al., 2019; Itokawa et al., 2022; Li et al., 2023). In the past, transcription factors (TFs), which are specialized proteins that can bind to specific non-coding DNA sequences (Rosenbauer and Tenen, 2007; Wang et al., 2021), such as enhancers, emerged as central players in controlling specific gene expression programs and the epigenetic landscape that drive normal hematopoiesis (Orkin, 1995; Lambert et al., 2018). Certain TFs yielded strong insights into the intracellular decision-making process, though these analyses were not especially comprehensive, as these focused on a limited number of TF pathways (Rothenberg and Göttgens, 2021). Today, with the comprehensive knowledge on TF networks, we can appreciate that very few TFs are strictly lineage-specific. Instead, we can now extract biologically meaningful insights from complex gene regulatory networks (GRNs) directed by combinations of sequence-specific TF bindings at regulatory DNA regions (Wilkinson et al., 2017). Furthermore, recent NGS technologies enable us to explore other regulatory layers of cell fate decision-making, including other molecules, such as the non-coding RNA sequences that are transcribed from enhancers (eRNAs) (Han and Li, 2022). While the importance of enhancers in driving cell-type specific gene expression has been known for decades, the role of these newly identified molecules in hematopoiesis is still elusive.

In this review, we discuss how current technical advancements reveal a more comprehensive picture of normal and abnormal hematopoiesis, delving into the TF-enhancer regulatory mechanisms, eRNAs, single-cell technologies, and the use of human induced pluripotent stem cells (hiPSCs) as a valuable model system. In addition, we discuss the steps that are being taken towards more precise methods of defining disease entities and medicine approaches.

## 2 Transcription factor binding at enhancers controls hematopoiesis

**FIGURE 1:**
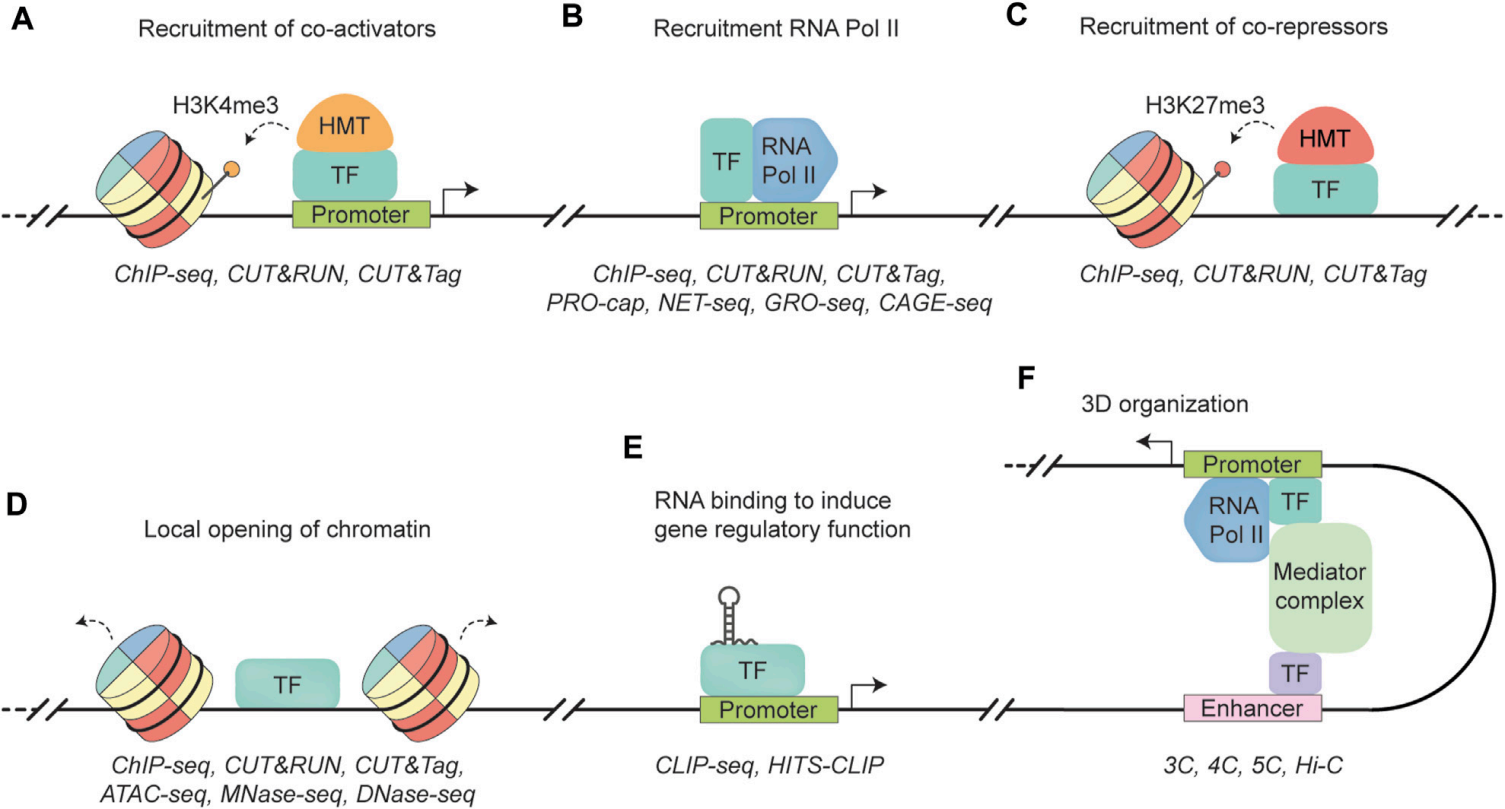
Schematic representation of transcription factor functionalities. **(A)** A transcription factor (TF) binds to a promoter region, recruiting a histone methyltransferase (HMT) to add three methyl groups to histone H3 lysine 4 (dotted arrow). This modification allows access to the genomic region, leading to transcription activation (black arrow). ChIP-seq, CUT&RUN, and CUT&Tag are well established techniques to quantify histone occupancy (Barski et al., 2007; Skene and Henikoff, 2017; Kaya-Okur et al., 2019). **(B)** A TF binds to the promoter region, recruiting RNA polymerase II (RNA Pol II) to facilitate transcription (black arrow). Similar to **(A)**, RNA Pol II occupancy can be quantified by ChIP-seq, CUT&RUN, and CUT&tag. In addition, techniques such as PRO-cap, NET-seq, GRO-seq, and CAGE-seq can map sites of transcriptionally active RNA Pol II by sequencing actively transcribed RNA molecules by RNA Poll II (Core et al., 2008; Churchman and Weissman, 2011; Takahashi et al., 2012; Kwak et al., 2013). **(C)** A TF recruiting a histone methyltransferase (HMT) that trimethylates lysine 27 in histone H3 (H3K27me3) (dotted arrow), leading to the formation of heterochromatin and gene repression. Similar to **(A)**, RNA Pol II occupancy can be quantified by ChIP-seq, CUT&RUN, and CUT&tag. **(D)** A TF that can recognize and bind to closed chromatin, leading to DNA accessibility (dotted arrow) and the establishment of cell-specific transcriptional programs. TF binding can be quantified by ChIP-seq, CUT&RUN, and CUT&tag. Methods to measure chromatin accessibility include ATAC-seq, MNase-seq, and DNase-seq (Albert et al., 2007; Boyle et al., 2008; Buenrostro et al., 2013). **(E)** TF interacts with RNA to regulate gene expression. Protein-RNA interactions can be measured with CLIP-seq methods, such as CLIP-seq and HITS-CLIP (Ule et al., 2003; Licatalosi et al., 2008). **(F)** A TF binds to an enhancer region to induce physical interaction between an enhancer region and gene promoter via the Mediator complex. As a result, enhancer-promoter looping facilitates the activation of gene transcription (black arrow). Techniques to measure these 3D interactions include the “Capturing Chromosome Conformation” techniques, such as 3C, 4C, 5C, and Hi-C (Dekker et al., 2002; Dostie et al., 2006; Simonis et al., 2006; Belton et al., 2012).

Blood cell development is a complex process that requires precise regulation of gene expression to ensure lineage-specific differentiation (Edginton-White and Bonifer, 2022). Key in this intricate orchestration are TFs, many of which were initially identified as being mutated in hematologic disorders (Prange et al., 2014). For instance, abnormal TFs take center stage in the development of most acute leukemias (Jutzi et al., 2019; Ottema et al., 2021), with a significant proportion of them being oncofusion proteins as a consequence of non-random chromosomal translocations (Martens and Stunnenberg, 2010; Marneth et al., 2018). Also, in cell fate determination, the expression of specific TF isoforms plays a crucial role (Clien et al., 2014). This is exemplified by the short isoform of NFIB (NFIB-S), which can regulate megakaryocyte maturation, whereas its long canonical counterpart cannot. Rather than acting in isolation, most TFs can form complexes with other proteins, allowing them to bind DNA directly or indirectly, depending on specific (co-)factors and environmental conditions (Daniel et al., 2020; Mitsis et al., 2020). TF binding can enable diverse mechanisms, including (a) recruitment of co-activators to introduce activating histone modifications (e.g., H3K4me3 or H3K27ac), or (b) recruitment of RNA polymerase II (Wright et al., 2006; Kaikkonen et al., 2013; Dhar et al., 2018), thus facilitating gene transcription (Figures 1A, B); (c) recruitment of co-repressors to induce repressive histone modifications (e.g., H3K27me3) (Boros et al., 2014), leading to chromatin compaction and gene silencing (Figure 1C); (d) directly binding to inaccessible DNA regions, displacing histones and creating an accessible binding landscape for other TFs (Figure 1D) (Magor et al., 2022); (e) interaction with RNAs to exert gene regulatory function (Figure 1E) (Oksuz et al., 2023); or (f) mediating three-dimensional (3D) organization of DNA, bringing distal DNA elements into physical proximity of proximal elements (Figure 1F) (Kim and Shendure, 2019). A TF’s mechanism often extends beyond just a single function. For instance, the hematopoietic TF SPI1 (PU.1) acts not only as a critical factor in opening condensed chromatin regions (Ungerbäck et al., 2018), but also partners with GATA1 to establish repressive chromatin states (Stopka et al., 2005). Likewise, CEBPA exhibits diverse roles, including direct transcriptional activation and interaction with histone modifiers to ensure normal myelopoiesis (Friedman, 2015). Another example is GFI1, an important TF in early hematopoiesis, which recruits the histone demethylase KDM1A (LSD1) to repress transcription by compacting chromatin (van Bergen and van der Reijden, 2019). Epigenomic regulation—including mechanisms such as transcription factors recruiting histone modifiers or DNA methylation patterns that repress TF binding (Héberlé and Bardet, 2019)—plays a vital role in establishing normal developmental pathways as well as contributing to leukemogenesis (Lin et al., 1998; Adams et al., 2012). The paradigms of this field has been well-documented in many reviews (Jaenisch and Bird, 2003; Stricker et al., 2016; Chen et al., 2017; Verma et al., 2021; Yang et al., 2023; Zhao et al., 2023).

The regions at which TFs can bind include core promoters and promoter-proximal elements, as well as long distance elements (Lenhard et al., 2012), such as enhancer (Bulger and Groudine, 2011; Prange et al., 2014), silencers (Doni Jayavelu et al., 2020), and insulators (Gaszner and Felsenfeld, 2006). Generally, for TFs to bind to their DNA elements, the chromatin structure needs to be made accessible. To overcome this restriction, specific “pioneering” TFs can directly bind to condensed chromatin to facilitate accessibility for other TFs to execute their function (Iwafuchi-Doi and Zaret, 2014; Lambert et al., 2018). These regulators are associated with initiating cell differentiation and activation of cell-specific genes, such as during cell reprogramming. For example, SPI1 and CEBPA/B expression can induce macrophage differentiation, and combined expression of KLF4, OCT4, SOX2, and MYC can generate iPSCs from human fibroblasts (Takahashi et al., 2007; Feng et al., 2008; Lowry et al., 2008). Among the non-coding DNA elements, enhancers and their associated TF binding are especially relevant for cell type-specific gene expression activation (Heinz et al., 2015; Cai et al., 2020). During development, enhancers can undergo progressive modifications that are initiated by successive waves of TF activity and chromatin remodeling (Spitz and Furlong, 2012). For example, a crucial step-wise PU.1 (mouse homolog of SPI1) occupancy event has been identified in the FIRE enhancer region, which plays an important role in myeloid differentiation (Krysinska et al., 2007). The process begins with PU.1 binding to the FIRE enhancer, subsequently triggering Egr-2 enhancer occupancy. Following these initial events, binding of PU.1 and other TFs, including C/EBPβ and Runx1, becomes possible, ultimately leading to increased levels of RNA polymerase II at the respective promoter region. It is proposed that long-range regulators, such as enhancers, and the physical relationship between their respective elements cannot be broken without having detrimental effects (Mongin et al., 2009). In contrast, less complex regulatory regions, such as those governing housekeeping genes, often exhibit higher susceptibility to evolutionary rearrangements and may be less affected by genome rearrangements. Instead, long-range interactions exert important pressure to preserve these interactions during vertebrate evolution, and, indeed, most are associated with conserved *cis*-regulatory elements and genes involved in development (Vavouri et al., 2006).

It is this precise coordination between TFs, cofactors, and genomic regions that controls the expression of a particular set of genes, ultimately defining the function and identity of a cell. Therefore, understanding TF-mediated gene expression regulation, as well as deregulation in case of mutation, provides valuable insights into essential biological processes, such as lineage determination, stress response, and maintenance of homeostasis. For example, important contributors to acute leukemias are chromosomal translocations involving TFs. An example of this are acute leukemias with *KMT2A* (*MLL*) gene rearrangements, which are responsible for approximately 10% of adult human leukemias (Krivtsov and Armstrong, 2007). Infant leukemias, exhibit an even higher incidence rate of *MLL* rearrangements, occurring in 70%–80% of cases (Mann et al., 2010; Meyer et al., 2013). More than 80 different protein partners have been described, with MLLT3 (AF9), MLLT1 (ENL), and AFF1 (AF4) being the most commonly observed fusion partners (Krivtsov and Armstrong, 2007; Winters and Bernt, 2017). Many of these fusion proteins play a role in regulating transcriptional elongation and directly or indirectly recruit the H3K79 histone methyltransferase DOT1L (Bitoun et al., 2007; Mueller et al., 2007; Li et al., 2014), leading to the deregulation of the epigenome. Indeed, epigenome deregulation is a common aberrancy in hematological diseases. This is exemplified by frequent mutations in epigenetic genes in hematopoietic malignancies, including chromatin regulators like histone methyltransferase EZH2 (Stasik et al., 2020), isocitrate dehydrogenase (IDH) genes (*IDH1* or *IDH2*) (Issa and DiNardo, 2021), and genes involved in DNA methylation, such as *TET2* and *DNMT3A* (Ley et al., 2010; Wouters and Delwel, 2016; Tulstrup et al., 2021). Although the specific recruitment of these enzymes to chromatin regions is not always clear, similar to MLL, for several, a direct link with TF binding has been reported. For example, GFI1B has been shown to interact and recruit the histone demethylase LSD1 to regulatory regions important for megakaryocyte development (Van Oorschot et al., 2019).

The affinities of TF binding to DNA sequences can vary—their so-called binding motif set (Boeva, 2016). Specific motif arrangements can be defined into two properties: motif composition and motif positioning. Motif composition refers to the presence of TF-specific binding motifs in the regulatory region to which TFs can bind. Motif positioning refers to the relative order, orientation, and spacing of TF motifs within a regulatory region. This positioning can promote cooperative protein-protein interactions and the recruitment of cofactors and transcriptional machinery (Spitz and Furlong, 2012). Cooperative TF interaction often results in changes to their binding specificity, resulting in significantly different affinity to composite sites compared to the individual TF’s motif (Jolma et al., 2015). By leveraging chromatin accessibility data followed by motif scanners, binding motifs can be used to predict TF binding (Van Heeringen et al., 2011). For example, when examining binding sites of the leukemogenic fusion protein RUNX1-RUNX1T1 (AML1-ETO), motif analysis uncovered a strong enrichment of the ETS factor core motif GGAAG. Subsequently, ERG/FLI1 were identified to facilitate RUNX1-RUNX1T1 binding, while also serving as transcriptional regulators of the oncofusion protein (Martens et al., 2012). Although predicting TF binding remains a challenging task, substantial efforts have been directed towards it as it can provide valuable insights into TF-mediated regulatory mechanisms (van der Sande et al., 2023). Recent developments in TF binding motif prediction have revealed the superiority of deep neural networks (DNNs) over traditional models (Alipanahi et al., 2015). Exploiting informative patterns from both DNase-seq coverage and DNA sequences plays a crucial role in achieving these accurate predictions (Chen et al., 2021).

TF binding prediction can be taken a step further by incorporating a multi-omics approach to infer TF importance during cell fate determination. Take, for instance, ANANSE (ANalysis Algorithm for Networks Specified by Enhancers) (Xu et al., 2021), which first predicts TF binding profiles using enhancer-specific sequence features and activity. Then, enhancer-based GRNs are determined by integrating the predicted TF binding profiles with gene expression data, and, lastly, TFs are ranked based on inferred importance through a differential network analysis between two types of cells. These superior prediction algorithms often rely on multiple data features, which can necessitate multiple experimental setups to gather the required data. However, improved understanding of molecular regulatory mechanisms offers potential solutions to this challenge. For example, by using Cap Analysis of Gene Expression (CAGE), unidirectional transcripts provide information on gene expression, while enhancer-specific non-coding RNAs (eRNAs) and their associated genomic regions marked by bidirectional transcription allows investigation of regulatory DNA regions and enhancer activity (Melgar et al., 2011; Sartorelli and Lauberth, 2020). This ANANSE-CAGE implementation allows to leverage differential GRNs and achieve high prediction performance while minimizing the need for extensive experimentation (Heuts et al., 2022). As experimental techniques and computational methods continue to advance, we anticipate innovative, multi-omics integration approaches to construct comprehensive regulatory networks and improve on the current TF prediction ability. Together, these are strategies that can enhance our understanding of hematopoietic systems and molecular mechanisms of disease.

## 3 Enhancer-derived RNAs as an important regulatory layer

With recent advancements in sequencing techniques and computational analyses, a growing number of non-coding RNAs (ncRNAs) have been discovered, including, amongst others, long noncoding RNAs (lncRNAs), enhancer RNAs (eRNAs), circular RNAs (circRNAs), and various small ncRNAs such as transfer-RNA (tRNA), small nucleolar RNA (snoRNA), and ribosomal RNA (rRNA) (Sartorelli and Lauberth, 2020; Sun and Chen, 2020; Suzuki, 2021; Huang et al., 2022; Hori et al., 2023). These ncRNAs play diverse roles in development, regulating transcription, RNA processing, chromatin state, and translation (Sun and Chen, 2020). Many ncRNAs display tissue-specific expression patterns and contribute to disease, including cancer. For instance, the lncRNA LAMP5-AS1 is implicated in *MLL* rearranged leukemia by promoting higher levels of H3K79 methylation, thereby influencing the self-renewal program and differentiation block (Wang et al., 2020). Among the ncRNAs, eRNAs hold significant potential in unraveling regulatory mechanisms in hematopoietic systems and pathobiology (Wang and Tang, 2021; Wan et al., 2022). However, their function remains a subject of controversy.

Most eRNA transcripts exhibit a 5′ cap structure, lack splicing, are non-polyadenylated and exhibit a short lifespan. Typically, these non-polyadenylated eRNAs are transcribed in a bidirectional manner (2D-eRNA) and range from 0.5 to 2 kb in length (Andersson et al., 2014). Additionally, there are unidirectionally transcribed polyadenylated eRNAs (1D-eRNA), which are generally longer (>4 kb) (Koch et al., 2011), although this latter group might comprise a mixture of true enhancer-templated RNAs and multi-exonic lncRNAs. Distinguishing between these two transcript types is not always straightforward, and they can be confused in the literature. Therefore, a clear working definition to distinguish the various enhancer-associated RNAs might be of great help to elucidate their function. For example, classifying eRNA molecules based on their length, polyadenylation, strand-specificity, and sequence-specificity might facilitate a more refined functional interpretation.

In general, there has been an abundance of evidence highlighting the link between eRNAs and active enhancers, revealing their association with increased transcription of neighboring genes (Wan et al., 2022). In many instances, transcription at enhancers precedes the transcription of associated genes (Arner et al., 2015). Interestingly, the levels and directionality of eRNA transcription appear to reflect the degree of enhancer activity, suggesting that they may serve as a more reliable predictor of enhancer activity compared to histone modifications and TF binding profiles at enhancers (Andersson et al., 2014; Henriques et al., 2018; Mikhaylichenko et al., 2018), even though these transcripts are inherently instable.

Over the past decade, the function of some of these eRNAs has been brought to light (Mousavi et al., 2013; Rahnamoun et al., 2018). Although both strands of the enhancer are often transcribed, the focus has predominantly been on unraveling the molecular function of one strand, leading to challenges in generalizing the functions of eRNAs, particularly as strand-specific eRNAs may share functional similarities with lncRNAs (Ørom et al., 2010). Nevertheless, several proposed mechanisms have emerged: eRNAs may act as “TF trapping” elements, enhancing TF binding or prolonging their residency time (Sigova et al., 2015); others have been implicated in facilitating loop formation by interacting with specific subunits of the cohesin complex, such as SMC3 and RAD21 (Li et al., 2013), thereby engaging the Mediator protein complex (Kagey et al., 2010; Lai et al., 2013); some eRNAs are thought to modulate the enhancer occupancy of the chromatin reader BRD4 by directly interacting with its tandem bromodomain (Rahnamoun et al., 2018); depletion of eRNAs during myogenesis has been observed to decrease DNase I accessibility (Mousavi et al., 2013), suggesting their involvement in establishing chromatin accessibility, although the specific chromatin remodeling complex responsible remains unknown; proximal to CBP binding sites, eRNAs have been found to interact with CBP in *cis*, resulting in localized acetyltransferase activity at enhancer, consequently, increasing H3K27ac and H3K18ac levels, and ultimately shaping the chromatin environment at target genes and fine-tuning transcriptional output (Bose et al., 2017). It is important to note that these CBP-eRNA interactions occur at multiple CBP-chromatin-binding loci, indicating sequence independent interaction. It is worth mentioning again that the examination of these interactions has primarily focused on a strand-specific manner, leading to ambiguity regarding the transcript type (1D- versus 2D-eRNA) (Quinn and Chang, 2015). Nevertheless, in the latter example, the sequence-independent nature of these interactions implies that the functional specificity of some eRNAs is driven by the location of eRNA transcription rather than their specific sequence.

**FIGURE 2:**
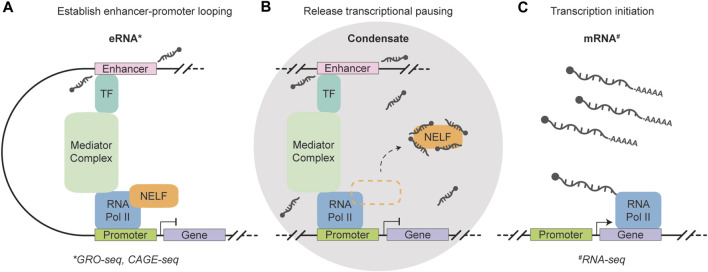
Schematic representation of the function of short, non-polyadenylation, strand- and sequence-independent enhancer RNAs. **(A)** A transcription factor (TF) recognizes and binds to the enhancer region, which triggers the transcription of eRNAs and facilitates enhancer-promoter looping through the Mediator complex. 5′-end capped eRNAs are released without including their polyadenylation. **(B)** A condensate is formed that houses eRNAs, RNA Polymerase II, and other pausing factors to enable efficient transcription regulation. The eRNAs function as a decoy for negative elongation factor (NELF) complexes and abolishes promoter-proximal pausing. **(C)** Enhancer-controlled transcription initiation generates mRNAs to shape cell type-specific gene expression. The eRNAs can be measured using GRO- and CAGE-seq (marked by the asterisk symbol) (Core et al., 2008; Takahashi et al., 2012), while RNA-seq can be used to quantify polyadenylated mRNA molecules (marked by the hash symbol) (Wang et al., 2009).

Recently, genome-wide *in vivo* evidence established 5′-end capped eRNAs with non-polyadenylated 3′-ends as important players in RNA Pol II pause release (Gorbovytska et al., 2022). Notably, these eRNAs do not rely on common structural or sequence motifs for their function. Instead, eRNAs with a length longer than 200 nucleotides and containing unpaired guanosines establish multiple allosteric contacts with NELF subunits -A and -E, triggering efficient release of the negative elongation factor (NELF). Moreover, the binding sites of eRNAs on NELF-A coincide with the sites of positive transcription elongation factor (P-TEFb) phosphorylation, suggesting that eRNA interaction with NELF may circumvent the need for P-TEFb activity in Pol II pause release. The recruitment of p-TEFB has been associated with common fusion partners of MLL, such as AF4 (AFF1) and AF9 (MLLT3) (Basu et al., 2020), making the protein, as well as associated eRNAs, putative important players in *MLL* translocation leukemias. A recent *in vitro* study has provided compelling evidence that eRNAs transcribed from super-enhancers exert influence over condensate formation by the purified Mediator complex (Henninger et al., 2021). This implies that the coexistence of eRNAs, Pol II, and other pausing factors within the same transcriptional condensate greatly enhances their ability to regulate gene expression, potentially by abolishing promoter-proximal pausing. Notably, eRNAs have been identified as decoys for NELF complexes (Schaukowitch et al., 2014), establishing a foundation for efficient transcriptional bursting (Figure 2). Comprehensive transcriptome-wide analyses have revealed the crucial role of enhancers in controlling burst frequencies, with changes in burst frequencies primarily shaping cell-type-specific gene expression patterns (Larsson et al., 2019). The Integrator complex, which is recruited by Pol II holoenzymes, plays a crucial role in terminating transcription and releasing 5′-end capped eRNAs without inducing their polyadenylation. This process greatly facilitates the rapid biogenesis of eRNAs that are essential for stimulus-dependent cellular processes, including differentiation (Lai et al., 2015). Interestingly, INTS3, a member of the integrator complex, is commonly mis-spliced across subtypes of AML, while not being present in healthy blood cells or other cancer types (Yoshimi et al., 2019). Further efforts to identify this mechanism may, therefore, hold significant value in understanding and targeting leukemia with integrator loss.

Taken together, 5′-end capped eRNAs with non-polyadenylated 3′-ends are suggested to facilitate a genome-wide sequence-independent mode of action that enables cell-type-specific gene expression. Increased abundance of these 2D-eRNA transcripts are suggested to facilitate efficient RNA Pol II pause release, likely controlling transcriptional burst kinetics. Importantly, the current definition of eRNAs can be confusing, leading to instances where certain eRNAs are attributed with strand- and/or sequence-specific functions. It is important to note that some of the cases in literature do not rule out the possibility that certain eRNAs may indeed be polyadenylated lncRNAs (Rahnamoun et al., 2018), whereas others clearly classify them as lncRNAs associated with enhancers (Lai et al., 2013). Despite this ambiguity, the transcription of eRNAs represents a crucial regulatory layer in the development of distinct cell types. It has been a well-established notion that investigation of the cell’s constituents, such as RNA molecules, contributes to our understanding of hematopoiesis and leukemia. Today’s technological advancements not only deepen our understanding of cellular identities in bulk, but also hold intriguing potential for investigating these identities at an unprecedented single-cell level.

## 4 Single-cell genomics redefines the hematopoietic model

Identifying blood cell types has been a major pursuit in the field of hematology, employing flow cytometry as the cornerstone for routine clinical diagnostics and the isolation of live cell populations with precision (Lin and Goodell, 2011; Van Dongen et al., 2012; Weeda et al., 2022). These methods rely on predefined sets of surface markers and gating strategies, leading to the identification of populations that are marked by dramatic functional and phenotypic differences. Expanding the repertoire of surface markers has led to increasingly intricate models of hematopoiesis (Cheng et al., 2020), requiring revisions in the understanding of lineage commitment decisions. Despite the indispensability of cytometry experiments in immunology, stem-cell biology, and hematology, gating schemes often still yield impure or heterogeneous populations (Paul et al., 2015; Velten et al., 2017), falling short of capturing the full complexity inherent in biological systems. The advent of single-cell sequencing technologies has ushered in a new era in the study of hematopoiesis and blood cell identification (Liggett and Sankaran, 2020; Campillo-Marcos et al., 2021). These cutting-edge techniques unlock the study of genetic, epigenetic, transcriptional, and proteomic landscapes at the individual cell level (Campillo-Marcos et al., 2021), revealing more diversity of blood cell types than previously recognized. For instance, by performing deep single-cell RNA sequencing and unbiased cell population clustering using healthy human peripheral blood mononuclear cells (PBMCs), six dendritic cell and four monocyte populations were revealed. These populations include a previously unknown DC subset that shares characteristics with plasmacytoid DCs (pDCs) and possesses the ability to activate T cells (Villani et al., 2017). Moreover, two novel monocyte types were identified, i.e., Mono3 and Mono4. Genes associated with cell cycle, differentiation, and trafficking were highly expressed in Mono3, while Mono4 exhibits high levels of cytotoxic gene signatures, including NK and T cell activation genes (Villani et al., 2017; Dutertre et al., 2019).

Single-cell strategies do not only provide the ability to deconvolute cellular heterogeneity, but also enable the determination of cell trajectories and lineage commitment (Loughran et al., 2020). Differentiation dynamics in adult hematopoiesis were unveiled as a continuous process (Karamitros et al., 2017; Velten et al., 2017; Buenrostro et al., 2018), diverging from the initial stepwise progression concept. For example, lineage-restricted cells emerge directly from a continuum of low-primed, undifferentiated hematopoietic stem and progenitor cell (HSPC) transitory states, rather than from discrete and definable intermediate progenitors (Velten et al., 2017). Single-cell sequencing also helped to define the molecular mechanisms important for early hematopoietic development. For example, by employing an hiPSC differentiation system to mimic endothelial-to-hematopoietic transition (EHT)—a process that is typically challenging to observe directly in humans—unveiled cell cycle regulators CDK1 and CDK4/6 as key drivers of process (Canu et al., 2020). Moreover, these cell cycle regulators were also found to be essential for HPC differentiation. In another study, an *in vitro* hiPSC system was combined with artificial neural networks (ANNs) trained on human fetal liver cells. This approach led to the identification of a range of hiPSC-derived HSPC phenotypes, including cells that exhibited a high transcriptional similarity to their fetal liver *in vivo* counterparts (Fidanza et al., 2020). The high level of similarity suggests that the limitations observed in hiPSC-derived HSCs could potentially be attributed to posttranscriptional mechanisms. Furthermore, single-cell RNA sequencing has enabled to extend these studies towards HSCs development in a human embryo (Popescu et al., 2019; Zeng et al., 2019). Together, the progress in this field has led to an expansion of our current understanding of hematopoietic development as well as a fundamental rethinking of our conventional models of hematopoiesis, but also diseases such as AML.

For the latter, single-cell genomics has been used to investigate clonal heterogeneity of AML and identifying specific sets of driver mutations (Morita et al., 2018). The order in which these mutations occur and lead to disease has been subjected to further investigation (Gawad et al., 2014; Miles et al., 2020). For example, a study with 146 samples from 123 patients demonstrated that AML is dominated by a small number of clones frequently harboring co-occurring mutations in epigenetic regulators, such as *DNMT3A* and *IDH1/2* (Miles et al., 2020). In contrast, distinct subclones frequently exhibit mutations in signaling genes, such as in the MAPK/ERK pathway, consistent with increased clonal diversity. By mapping clonal trajectories for each sample, synergistic combinations of mutations such as *NPM1–FLT3-ITD* or *DNMT3A–IDH2* were shown to promote clonal expansion and dominance. Single-cell sequencing also allows for the examination of clone resistance to therapeutic interventions (Giustacchini et al., 2017; Demaree et al., 2021; Triana et al., 2021). Furthermore, comprehensive exploration of transcriptomic and epigenomic heterogeneity, as well as capturing proteogenomic states in leukemia has provided a valuable strategy for cell type characterization (Litzenburger et al., 2017; van Galen et al., 2019). Overall, in the context of disease, single-cell sequencing enables us to gain insights into the pathogenesis of leukemic transformation and the evolving complexity of clonal dynamics during disease progression.

Even though this field is rapidly moving forward, there are still major hurdles that need to be faced. First and foremost, most of these techniques come with cost-intensive setups, including the need for large data storage and computer processing power (Angerer et al., 2017). In addition, single-cell technologies generate massive amounts of data that suffers from high sparsity and methodological noise which requires context-dependent quality control and processing (Lähnemann et al., 2020). Any single-cell sequencing technique is subjected to complex integrating strategies. Statistical and computational methods require both adaptability and stringency to deal with varying levels of resolution, uncertainties inherent to measurements and their accurate quantification during analysis, and the ability to scale single-cell methodologies to accommodate for differences in samples with more cells, increased feature measurements, and broader coverage. Furthermore, as new methods, algorithms, and analysis tools continue to emerge, comprehensive gold-standards become a moving target. It is crucial to benchmark novel tools systematically, ensuring they can consistently generate expected results based on these gold-standards (Weber et al., 2019; Heumos et al., 2023). These tools are required to effectively handle high levels of sparsity, noise, and technical biases. The review of Lähnemann et al. (2020) provides an in-depth discussion highlighting these recurring themes and challenges (Lähnemann et al., 2020). For now, a comprehensive best-practice workflow has been established, comprising of independently benchmarked computational methods. The workflow typically involves several common steps: (I) data preprocessing, which encompasses tasks such as normalization, integration, and dimensionality reduction, along with quality control visualization; (II) identification of cellular structure, involving techniques like clustering, annotation, and trajectory inference; and (III) uncovering molecular mechanisms, including analyses such as differential expression, TF activity, and gene regulatory networks. To delve deeper into this subject, we refer to the best-practice workflows outlined by Heumos et al. (2023). The significant impact of single-cell technologies in reshaping conventional understanding of hematopoiesis underscores the crucial role of data-driven exploration in understanding the immune system and disease. Exploiting these methods in conjunction with human differentiation systems, such as hiPSCs-derived blood differentiation platforms, emerged as a compelling strategy to scrutinize the precise mechanisms governing cell fate determination and disease mechanisms.

## 5 Human induced pluripotent stem cells unlock avenues for disease modeling and regenerative medicine

Novel experimental model systems, such as the hiPSC differentiation model mentioned above, have played a vital role in providing invaluable insights into the development and function of the human immune system. Before these became available, our understanding of early mammalian hematopoiesis has predominantly relied on studies conducted in small animal models due to ethical concerns surrounding research involving human fetal tissue. Mice have been key in our understanding of immunology (Masopust et al., 2017). Despite considerable homology, notable physiological and genetic differences exist between the innate and adaptive immune systems of humans and mice. Through genetic modification and immunosuppression of mice, including the overexpression of human growth factors, “humanized mice” have enabled significantly enhanced engraftment after xenotransplantation (Stripecke et al., 2020). These models offer a relevant *in vivo* context by recapitulating the evolutionary specialization and diversity of genotypic and phenotypic traits seen in humans (Cogels et al., 2021; Mian et al., 2021; Liu et al., 2023).

Our understanding of hematopoiesis dates back over a century to the discovery of hematopoietic stem cells (HSCs), which initiated the paradigm that a single cell could be the precursor of all blood cells (Ramalho-Santos and Willenbring, 2007). Presently, we recognize HSCs to stand at the apex of the definitive and adult hematopoietic hierarchy, possessing two key characteristics: differentiation into all mature blood lineages and self-renewability to maintain the hematopoietic landscape (Mann et al., 2022). The exploitation of these aspects holds tremendous promise for therapeutic interventions aimed at restoring the hematopoietic landscape through a series of myeloid, lymphoid, and erythroid intermediates (Fung et al., 2003; Yanada et al., 2006; Angelucci et al., 2014). Indeed, HSC isolation from donors has immense therapeutic potential and is also a critical tool for advancing stem cell research. Unfortunately, donor variability can impact the quantity and quality of HSCs isolation (Belderbos et al., 2020). Alternatively, human iPSCs have garnered particular interest for establishing a renewable source of hematopoietic cells without genetic background variability (Demirci et al., 2020). A pluripotent cell state is acquired through TF-induced reprogramming of adult somatic cells, typically OCT4, SOX2, KLF4, and MYC (Takahashi et al., 2007; Lowry et al., 2008). Somatic cells can be obtained from a donor through a blood sample, allowing the generation of patient-derived iPSCs (Kotini et al., 2023). Donor-specific reprogramming mitigates donor variability inherent to blood cell harvests and enables context-dependent investigation into patient-specific properties and genetic information. The conventional method of inducing hematopoiesis from hiPSCs follows a stepwise differentiation protocol that emulates the natural developmental pathway of early hematopoietic cells. This process involves initiating cell fate through stage-specific mixtures of cytokines, commencing with the formation of mesodermal cells and subsequently progressing to the generation of HSPCs, culminating in the production of late-stage hematopoietic cells (Mandoli et al., 2016; Walasek et al., 2017). The iPSC-derived HSCs share phenotypic similarities with multipotent HSCs derived from the definitive wave, though generally display limited engraftment capacities (Demirci et al., 2020). Higher engraftable HSCs can be achieved through genetically modifying the iPSCs followed by transient TF expression (Tan et al., 2018), though this is paired with higher leukemic potential, which requires further attention. Alternatively, HSC-like cells can be derived *in vitro* through the direct conversion of PSCs or adult endothelial cells by inducing the expression of TF-encoding genes (Lis et al., 2017; Sugimura et al., 2017). The latter approach demonstrated the ability to produce self-renewing HSCs with long-term, clonally-derived engraftment capabilities that exhibited *bona fide* HSC characteristics. This differentiation pathway involved the co-culturing mouse endothelial cells with angiocrine factors derived from the vascular niche. The arterial program is frequently overlooked when employing a hiPSC system, potentially resulting in produced HSCs that correspond to transient definitive hematopoiesis from the yolk sac, lacking lymphopoiesis. Integrating an arterial program into the hiPSC differentiation process can therefore contribute to the advancement of definitive hematopoiesis (Park et al., 2018).

Recent advancements in genetically modifying hiPSCs unlocked exciting avenues for disease modeling, drug discovery, and regenerative medicine (Gähwiler et al., 2021; Tu et al., 2021; Heuts et al., 2023). These advancements provide a platform for studying hematopoietic cells that, in some cases, are challenging to genetically manipulate without triggering an immune response. This system allows for nearly limitless cell growth and single-cell clonal expansion (Singh, 2019), thus providing a reliable cell source for studying genetically engineered hematopoietic cells. For instance, by integrated doxycycline dose-dependent oncofusion gene expression, e.g., KMT2A-MLLT3 (MLL-AF9) or RUNX1-RUNX1T1 (AML1-ETO), AML programs can be identified in the context of *in vitro* iPSC-derived hematopoietic differentiation (Tijchon et al., 2019; Heuts et al., 2023). Thus, providing insights into blood development and disease mechanisms. Furthermore, patient-derived iPSC differentiation can recapitulate the pathogenesis of leukemia patients, even after reprogramming (Taoka et al., 2018). When combined with humanized mouse models, this makes for a powerful strategy. For example, the combination of humanized mice and patient-derived iPSCs enabled the study of how patient-derived immune cells interact with their own tumor cells, offering valuable insights into the effectiveness of autologous cancer immunotherapies (Moquin-Beaudry et al., 2022). Another study leveraged the use of chronic myelomonocytic leukemia (CMML) patient-derived iPSCs to establish a humanized CMML mouse model (Taoka et al., 2018). By using this system clinically relevant drug candidates were tested, leading to the identification of liposomal clodronate as a potential therapeutic agent for CMML treatment.

Unfortunately, iPSC research faces lengthy, labor-intensive, and costly processes requiring specialized expertise. Despite the potential of using iPSCs for cell replacement therapies, the realization of this potential has been hindered by properties inherent to iPSCs, including tumorigenicity, immunogenicity, and heterogeneity (Yamanaka, 2020). Fortunately, there are ongoing efforts to address these challenges. One such approach involves to use of microfluidic chips (organs-on-chips) to culture human tissues with patient-derived cells and immune cells, thus creating a more human-like microenvironment for assessing tumorigenicity (Sato et al., 2019). Another approach considers a fatal gene approach to selectively eliminate immature proliferating cells, effectively preventing the formation of tumors (Kojima et al., 2019). There are also ongoing efforts to identify factors that can reduce heterogeneity or serve as markers for undifferentiated iPSCs (Kunitomi et al., 2016; Sekine et al., 2020). Furthermore, iPSC workflows are becoming more efficient and less biased. For instance, a label-free and non-invasive approach leveraging time-lapse imagery can be employed to measure morphological dynamics and guide the selection of iPSC colonies. This method enabled the detection of the earliest changes in iPSC colony formation as early as day 7, within the 20–24 day process (Fan et al., 2017). In addition, the processing and analysis of vast amounts of data can serve to sustain the use of iPSC derivatives as platforms for drug screening and, thus, contribute to our understanding of how drugs impact key cellular functions. For instance, by combining RNA data from an iPSC models with a K-nearest neighbors (k-NN) algorithm, researchers successfully identified eight small molecules from a panel of 1,595 molecules (Theodoris et al., 2021). These eight molecules demonstrated the ability to broadly correct dysregulated gene networks in patient-derived iPSCs associated with a common form of heart disease involving heterozygous loss-of-function NOTCH1 (N1) mutations, specifically affecting the aortic valve (AV). The most effective therapeutic candidate for GRN correction demonstrated its effectiveness by extending to primary aortic valve cells derived from over 20 patients with sporadic aortic valve disease, and it successfully prevented aortic valve disease in a mouse model. This approach proves to be an effective strategy for identifying potential therapeutic molecules, as it takes into account the broader GRNs underlying diseases, which are often overlooked. Altogether, these studies underscore the significance of hiPSCs as a model for investigating normal development, diseases, and therapeutic approaches. Refining these systems for early hematopoiesis and improved blood cell production will advance mechanistic studies, small molecule screening, and regenerative medicine.

## 6 Towards precision medicine

The growing availability of omics-data, along with our ability to integrate and analyze large and complex datasets, holds tremendous promise in enhancing patient care and improving outcomes. In the past, the introduction of whole-genome sequencing revolutionized the profiling of patients with AML or MDS (Duncavage et al., 2021). This groundbreaking technique outperformed traditional cytogenetic analysis, enabling more efficient and nuanced risk stratification. A significant outcome of this progress materialized in the International Consensus Classification (ICC) of myeloid neoplasms and acute leukemias (Arber et al., 2022), as well as the 2022 rendition of the World Health Organization (WHO) classification of hematolymphoid neoplasms and the European LeukemiaNet (ELN) risk stratification prerequisites (Döhner et al., 2022; Khoury et al., 2022). While patient characteristics, including age and fitness, have a significant impact on prognosis and treatment, there has been a growing emphasis on identifying specific genetic driver events. A deeper understanding of the underlying molecular mechanisms can facilitate innovative treatment methods. For example, in *MLL*-rearranged AML, the inhibition of histone methyltransferase DOT1L, which suppresses MLL-fusion-driven gene expression, only leads to complete remission in a limited number of patients. This suggests the presence of a mechanism that allows for sustained gene expression driven by MLL-fusion proteins, bypassing the rapid loss of epigenetic control (Stein et al., 2018). A combinatorial drug approach was employed, combining DOT1L inhibition with the inhibition of the MLL-Menin interaction, which resulted in significant enhancement of differentiation induction and cell killing in different models of MLL-related diseases, including primary leukemia cells (Dafflon et al., 2016). Importantly, this approach demonstrated selectivity towards leukemia cells with *MLL* rearrangements, while sparing normal hematopoiesis and leukemias without *MLL* rearrangements. These findings provide a compelling rationale for exploring novel combination drug therapies as a means to enhance treatment outcomes. By building upon the example on heart disease above (Theodoris et al., 2021), a GRN-based approach might also uncover additional drug combinations for MLL-driven leukemia.

GRN-based research holds also significant promise for enhancing existing diagnostic categories, treatment approaches, and facilitate innovative clinical trials. For instance, a GRN-based analysis identified sex-linked differences in colon cancer drug metabolism, suggesting that male and female tumor cells are programmed to respond differently (Lopes-Ramos et al., 2018). Targeting the drug metabolism pathway more effectively was associated with improved overall survival in females receiving adjuvant chemotherapy. Sex differences have also been identified in both AML incidence and overall survival, as well as in hematopoiesis (Nakada et al., 2014; Stabellini et al., 2023). This emphasizes the need to expand beyond our conventional methods and consider comprehensive aberrant GRNs as the foundation for intervention strategies (Weighill et al., 2021). The challenge lies in implementing this approach efficiently and cost-effectively within current workflows. Nevertheless, TF-directed GRNs can offer valuable insights into therapy response for complex (hematopoietic) diseases and might serve as a driving force for disease categorization.

## 7 Conclusion and future perspectives

With the rapid advancements in NGS techniques, fundamental concepts are being explored that are not only limited to normal and abnormal hematopoiesis. For example, we are long aware that TF-enhancer interactions are central in differentiation and cancer, however the 1D- and 2D-eRNAs that are associated with these regions are poorly understood. Further examination is required to address lingering questions regarding the mechanisms of eRNAs, including the examination of secondary structures, occurrence and function of RNA modifications [e.g., methylation by N6-adenosine (m6A)], involvement in higher-order chromatin organization, and in phase-separated condensates. Furthermore, the downregulation of these transcripts by antisense oligonucleotides (ASOs) enables the examination of specific signaling pathways that are impacted by eRNAs, even in the context of tumor growth (Zhao et al., 2016; Pan et al., 2021). Given the ambiguity between 1D- and 2D-eRNAs, functional studies might lead to misunderstandings, so a working definition that discriminates these transcript types based on, for example, their length, polyadenylation, strand-specificity, and sequence-specificity might be in order. However, this distinction might not be necessary for elucidating specific enhancer-promoter function as a potential novel therapy target. The next step might be to further investigate their role in regulating immune-related genes, and in normal and malignant hematopoiesis. The non-coding DNA regions that are associated with bidirectional enhancer transcription may be used to further help elucidate TF-directed cell fate and differentiation mechanisms, potentially expanding our repertoire of regulatory suspects that control normal and abnormal hematopoiesis.

Single-cell sequencing holds a wealth of data that could be valuable in approximating continuous relationships within the data. In order to gain an accurate and comprehensive view of the cellular composition of normal and disease samples, it is essential to integrate multiple omics data for one sample simultaneously, i.e., multimodality. By employing single-cell multimodal techniques, researchers may generate novel hypotheses or design mathematical algorithms for prediction tasks, such as drug sensitivity and efficacy, gene dependence prediction, and patient stratification. While promising, these techniques come with high costs and complex analysis challenges. The samples are faced with high sparsity and manifest differences in their size, format and dimensionality, noisiness, information content, and their mutual concordance, thus making it challenging to integrate (Wani and Raza, 2019). Deep learning methods are being explored and need to be further developed to tackle these challenges (Chaudhary et al., 2018). These approaches enable direct processing of sequencing data, eliminating the need for manual feature extraction by automatically learning relevant features from the data (Erfanian et al., 2023). Additionally, they have the capability to capture complex, non-linear relationships, and interaction effects that extend across different genomic scales. Unfortunately, acquiring the large amounts of data required for this purpose is not easily attainable. Despite this hurdle, an increased use of deep learning methods can facilitate a deeper understanding of complex (patho)biology.

Ultimately, integrating multi-omics data and enhancer regulatory networks can offer a more comprehensive and profound understanding of normal and abnormal hematopoietic development. Taking strides in this direction will provide invaluable novel insights in normal and abnormal hematopoietic development that can lead to improved therapeutic strategies.
